# Dual Repression of the Multidrug Eﬄux Pump CmeABC by CosR and CmeR in *Campylobacter jejuni*

**DOI:** 10.3389/fmicb.2016.01097

**Published:** 2016-07-13

**Authors:** Tara Grinnage-Pulley, Yang Mu, Lei Dai, Qijing Zhang

**Affiliations:** Department of Veterinary Microbiology and Preventive Medicine, College of Veterinary Medicine, Iowa State University, AmesIA, USA

**Keywords:** *Campylobacter jejuni*, transcriptional regulation, eﬄux pump, CmeABC, oxidative stress, CosR, CmeR

## Abstract

During transmission and intestinal colonization, *Campylobacter jejuni*, a major foodborne human pathogen, experiences oxidative stress. CosR, a response regulator in *C. jejuni*, modulates the oxidative stress response and represses expression of the CmeABC multidrug eﬄux pump. CmeABC, a key component in resistance to toxic compounds including antimicrobials and bile salts, is also under negative regulation by CmeR, a TetR family transcriptional regulator. How CosR and CmeR interact in binding to the c*meABC* promoter and how CosR senses oxidative stress are still unknown. To answer these questions, we conducted various experiments utilizing electrophoretic mobility shift assays and transcriptional fusion assays. CosR and CmeR bound independently to two separate sites of the *cmeABC* promoter, simultaneously repressing *cmeABC* expression. This dual binding of CosR and CmeR is optimal with a 17 base pair space between the two binding sites as mutations that shortened the distance between the binding sites decreased binding by CmeR and enhanced *cmeABC* expression. Additionally, the single cysteine residue (C218) of CosR was sensitive to oxidation, which altered the DNA-binding activity of CosR and dissociated CosR from the *cmeABC* promoter as determined by electrophoretic mobility shift assay. Replacement of C218 with serine rendered CosR insensitive to oxidation, suggesting a potential role of C218 in sensing oxidative stress and providing a possible mechanism for CosR-mediated response to oxidative stress. These findings reveal a dual regulatory role of CosR and CmeR in modulating *cmeABC* expression and suggest a potential mechanism that may explain overexpression of *cmeABC* in response to oxidative stress. Differential expression of *cmeABC* mediated by CmeR and CosR in response to different signals may facilitate adaptation of *Campylobacter* to various environmental conditions.

## Introduction

*Campylobacter jejuni* is a microaerophilic, Gram-negative pathogen causing foodborne enteritis in humans (Black et al., 1988). In some animal species, such as birds, *C. jejuni* is a commensal organism and is well adapted to the enteric environment (Butzler, 2004; Lin et al., 2005b). Colonization in the intestines requires *C. jejuni* to be resistant to the antimicrobial action of bile (Lin et al., 2003; Raphael et al., 2005). The CmeABC multidrug eﬄux pump plays a key role in bile resistance and is one of the known mechanisms required for intestinal colonization (Lin et al., 2003). CmeABC is a tripartite eﬄux system composed of the inner membrane protein CmeB, the periplasmic fusion protein CmeA, and the outer membrane protein CmeC (Lin et al., 2002). This three-gene operon is regulated by a TetR family regulator named CmeR (Lin et al., 2005a), which binds to a 16-base inverted repeat within the *cmeABC* promoter and inhibits the expression of *cmeABC* (Lin et al., 2005a). CmeABC is an important player for antibiotic resistance and is the predominant mechanism for bile resistance in *C. jejuni*, making it essential for intestinal colonization (Lin et al., 2002, 2003, 2005b). The expression of *cmeABC* is inducible by bile. The induction is initiated by bile binding to CmeR, which triggers a conformational change in the DNA-binding domain of CmeR, thereby releasing CmeR from the promoter and increasing the pump expression (Lin et al., 2005b; Lei et al., 2011).

As a microaerobic organism, *Campylobacter* is sensitive to atmospheric oxygen and to oxidative stresses from host immune systems including hydrogen peroxide produced by intestinal epithelium (Corcionivoschi et al., 2012). Hydrogen peroxide is one of the reactive oxygen species (ROS) that induce oxidative damage to cells (Dwyer et al., 2009; Chiang and Schellhorn, 2012). Other ROS include superoxide and hydroxyl radicals. Inducing oxidative stress by ROS is one mechanism the immune system employs to defend against pathogens (Chiang and Schellhorn, 2012). The orphan response regulator CosR is an oxidative stress response regulator in *C. jejuni*, modulating the expression of oxidative stress response and resistance genes including *katA, sodB*, and *ahpC* (Hwang et al., 2011, 2012). Interestingly, CosR also represses the expression of CmeABC by binding to a region in the *cmeABC* promoter containing a CosR binding site (Hwang et al., 2012). This suggests a link between the oxidative stress response and CmeABC antibiotic eﬄux system in *Campylobacter*. Regulation of antibiotic eﬄux pumps has been previously linked to oxidative stress in other bacteria (Poole, 2012a,b). For example, MexR of *Pseudomonas aeruginosa* senses oxidative stress through two cysteine residues (Chen et al., 2008). The reduced form of MexR serves as a repressor for the MexAB-OprM eﬄux pump, but once oxidized, MexR is dissociated from the promoter, leading to overexpression of MexAB-OprM (Poole et al., 1996; Chen et al., 2008). Collectively, these observations suggest that oxidative stress response and antibiotic eﬄux systems are intertwined in bacteria.

*Helicobacter pylori*, an organism previously classified as *Campylobacter*, contains a homolog of CosR named HP1043 (Müller et al., 2007). CosR can functionally substitute for HP1043 when expressed in *H. pylori* from the HP1043 promoter (Müller et al., 2007). HP1043 forms a dimer and contains two cysteine residues that modulate its regulatory function (Hong et al., 2007; Müller et al., 2007; Olekhnovich et al., 2013). CosR contains a single cysteine residue (C218) that corresponds to C215 of HP1043. Based on the HP1043 sequence and its crystal structure, the single cysteine residue of CosR likely resides in the dimer interface (Hong et al., 2007). It has been known that oxidation of cysteine residues at the dimer interface affects the conformation and function of regulatory proteins (Antelmann and Helmann, 2011; Dubbs and Mongkolsuk, 2012), but it is unknown if modification of C218 in CosR modulates its binding activities to promoter DNA.

Previous work suggested that CmeABC is also likely regulated by a CmeR-independent mechanism, because *cmeABC* was further induced by bile in the absence of CmeR (Lin et al., 2005b). The excess induction in the absence of CmeR was attributed to an unknown mechanism regulating *cmeABC* expression (Lin et al., 2005b). Additionally, our work studying various *cmeABC* promoter mutations further indicated that multiple regulators may bind to the promoter sequence of *cmeABC* (Grinnage-Pulley and Zhang, 2015). Our observations and the work of Hwang et al. (2012) on CosR binding to the promoter of CmeABC suggests that the regulation of *cmeABC* is complex and likely involves interaction of multiple regulators. Therefore, we hypothesize that CosR and CmeR function as a dual mechanism in modulating the expression of CmeABC and that C218 in CosR may serve as a sensor for oxidative stress. To test this hypothesis, we examined the roles of CosR and CmeR in the regulation of *cmeABC* as well as the role of C218 in modulating the function of CosR under oxidative stress.

## Materials and Methods

### Bacterial Strains and Growth Conditions

*Campylobacter jejuni* strains X7199 (Sahin et al., 2012), NCTC 11168 (Parkhill et al., 2000), 81–176 (Black et al., 1988), 81–176Δ*cmeR* (Lin et al., 2005a), and 11168Δ*cmeR* (Lin et al., 2005a) were used in this study (**Table 1**) and they were routinely cultured on Mueller Hinton (MH) agar or in MH broth (Difco, Detroit, MI, USA) at 42°C under microaerobic conditions (5% O_2_, 10% CO_2_, 85% N_2_). Media was supplemented with kanamycin at 30 μg/mL or chloramphenicol at 4 μg/mL as needed. *Escherichia coli* strains DH5α (Invitrogen), JM109 (Agilent Technologies), and DH5αpRK2013 (Miller et al., 2000) were routinely cultured at 37°C with Luria-Bertani (LB) broth or LB agar (Difco), which was supplemented with 30 μg/mL kanamycin or 100 μg/mL ampicillin when needed (**Table 1**).

**Table 1:** Bacterial strains used in this study.

Strains	Description	Source
*Campylobacter jejuni*		
NCTC 11168		Parkhill et al., 2000
11168*ΔcmeR*	Derivative of NCTC 11168, *cmeR::cat*	Lin et al., 2005a
11168W7pMW561	Highly motile variant of NCTC 11168 carrying pMW561	Guo et al., 2008
X7199	Human clinical isolate	Grinnage-Pulley and Zhang, 2015
81–176	Human clinical isolate	Black et al., 1988
81–176pMW10	Derivative of 81–176 carrying pMW10	Grinnage-Pulley and Zhang, 2015
81–176pMW11168	Derivative of 81–176 carrying pMW11168	Grinnage-Pulley and Zhang, 2015
81–176pMW81–176	Derivative of 81–176 carrying pMW81–176	Grinnage-Pulley and Zhang, 2015
81–176pMWX7199	Derivative of 81–176 carrying pMWX7199	Grinnage-Pulley and Zhang, 2015
81–176pMW561	Derivative of 81–176 carrying pMW561	This study
81–176*ΔcmeR*	Derivative of 81–176, *cmeR::cat*	Lin et al., 2005a
81–176*ΔcmeR* pMW10	Derivative of 81–176, *cmeR::cat* carrying pMW10	Grinnage-Pulley and Zhang, 2015
81–176*ΔcmeR* pMW11168	Derivative of 81–176, *cmeR::cat* carrying pMW11168	Grinnage-Pulley and Zhang, 2015
81–176*ΔcmeR* pMW81–176	Derivative of 81–176, *cmeR::cat* carrying pMW81–176	Grinnage-Pulley and Zhang, 2015
81–176*ΔcmeR* pMWX7199	Derivative of 81–176, *cmeR::cat* carrying pMWX7199	Grinnage-Pulley and Zhang, 2015
81–176*ΔcmeR* pMW561	Derivative of 81–176, *cmeR::cat* carrying pMW561	This study
*Escherichia coli*		
DH5α	F-Φ80*lac*ZΔM15 Δ(*lac*ZYA-*argF*) U169 *rec*A1 *end*A1 *hsd*R17 (t_κ_^-^m_κ_^+^) *pho*A *supE44*λ^-^ *thi*^-^*1 gyr*A96 *rel*A1	Invitrogen
DH5αpRK2013	Helper strain for conjugation	Miller et al., 2000
JM109	e14^-^(McrA^-^) *rec*A1 *end*A1 *gyr*A96 *thi-1 hsd*R17(*t*_κ_^-^*m*_κ_^+^) *sup*E44 *rel*A1 *Δ*(*lac*-proAB) [F’ *traD36 pro*AB *lacqZΔM15*]	Agilent
JM109pQE::*Cj0355c*	Derivative of JM109 carrying pQE::*Cj0355c*	This study
JM109pQE::*Cj0355c*652	Derivative of JM109 carrying pQE::*Cj0355c*652	This study
JM109pQECmeRSS	Derivative of JM109 carrying pQECmeRSS	Oakland et al., 2011
DH5αpMW561	Derivative of DH5α carrying pMW561	This study

### Recombinant CosR and CmeR Construction and Purification

Recombinant CosR was produced using the pQE30 plasmid (**Table 2**) expression system (Qiagen). Amplification of the *cosR* (*Cj0355c*) sequence from NCTC 11168 was performed with primers *Cj0355c*-F1 and *Cj0355c*-R1 (**Table 3**) for one cycle of 94°C for 5 min, 30 cycles of 94°C for 30 s, 50–55°C for 30 s (1°C temperature gradient), 72°C for 1 min, and 1 cycle of 72°C for 10 min followed by a hold at 4°C. This PCR product and the pQE30 plasmid were digested with *BamHI* and *KpnI* (Promega). The digested PCR product and pQE30 were purified using the QIAquick PCR purification and QIAprep Spin Miniprep kits (Qiagen), respectively. The vector and insert were then ligated with T4 DNA ligase (Roche) and transformed into *E. coli* JM109 via electroporation using a Gene Pulser Xcell per the manufacturer’s instructions (Bio-Rad Laboratories, Inc.). The transformants were selected on LB agar supplemented with ampicillin (100 μg/mL). The plasmid was purified from transformant JM109pQE::*Cj0355c* (**Table 2**) and was sequenced using the Type III/IV primer (Qiagen; **Table 3**) to confirm there were no mutations in the cloned *Cj0355c* gene. DNA sequencing was conducted by the Iowa State University DNA Sequencing Facility using an Applied BiosystemsAB3730xI DNA analyzer. The recombinant CosR, named rCosRWT, was induced and purified from JM109pQE::*Cj0355c* under native conditions as described in the manufacturer’s instruction (Qiagen). Following purification, the protein was desalted with a PD-10 desalting column (GE Healthcare) and the molecular weight was verified by SDS PAGE as described in Grinnage-Pulley and Zhang (2015).

**Table 2:** Bacterial plasmids used in this study.

Plasmids	Description	Source
pMW10	*E. coli* – *Campylobacter* shuttle vector carrying promoterless *lacZ*, KanR	Wösten et al., 1998
pMW11168	pMW10 carrying the *cmeABC* promoter from NCTC11168 fused to *lacZ*, KanR	Grinnage-Pulley and Zhang, 2015
pMW81–176	pMW10 carrying the *cmeABC* promoter from 81 to 176 fused to *lacZ*, Kan^R^	Grinnage-Pulley and Zhang, 2015
pMWX7199	pMW10 carrying the *cmeABC* promoter from X7199 fused to *lacZ*, Kan^R^	Grinnage-Pulley and Zhang, 2015
pMW561	pMW10 carrying the *Cj0561c* promoter fused to *lacZ*, Kan^R^	Guo et al., 2008
pQE30	Expression vector for N-terminal 6-His tagged proteins, Amp^R^	Qiagen
pQECmeRSS	pQE30 carrying CmeR with the C69S and C166S substitutions	Oakland et al., 2011
pQE::*Cj0355c*	pQE30 carrying *Cj0355c*	This study
pQE::*Cj0355c*652	pQE30 carrying *Cj0355c* with the T to A mutation at nt 652	This study
pUC57	Cloning vector, Amp^R^	Genscript
pUC57P14D	pUC57 with a 14 bp deletion in the *cmeABC* promoter from 81 to 176	This study

**Table 3:** Oligonucleotide primers used in this study^1^.

Primer	Sequence	Source
*Cj0355c*-F1	CGCGGGATCCAGAATTTTAGTTATAGAAGATGAG (BamHI)	This study
*Cj0355c*-R1	GCAGGGTACCTGTAAGATTTTTTAGGGAAGCAG (KpnI)	This study
CosR652-F	AGGATACCGTTTCAGCTTCCCTAAAAA	This study
CosR652-R	TTTTTAGGGAAGCTGAAACGGTATCCT	This study
GSF	CTAAATGGAATCAATAGCTCC	Lin et al., 2005a
GSR1	GCACAACACCTAAAGCTAAAA	Lin et al., 2005a
AF	AACCTCAAGTTAGCGGCGTA	Lin et al., 2005a
AR	AATCCTTGCTTGCATTTTCG	Lin et al., 2005a
Type III/IV	CGGATAACAATTTCACACAG	Qiagen

*^1^Restriction sites are indicated by underlined sequences.*

To mutate the single cysteine residue (C218) in CosR, pQE::*Cj0355c* was used as a template for site directed mutagenesis of *cosR.* Primers CosR652-F and CosR652-R (**Table 2**) were designed to introduce a T to A substitution at nucleotide 652, resulting in the replacement of cysteine residue 218 by serine. The QuikChange II Site-Directed Mutagenesis kit (Agilent Technologies) was used to introduce the mutation by one cycle of 95°C for 30 s followed by 16 cycles of 95°C for 30 s, 55°C for 30 s, and 68°C for 4 min. The amplified product was cooled on ice for 2 min before *Dpn*-I digestion of parental DNA at 37°C for 1 h. The product, pQE::*Cj0355c*652 (**Table 3**), was then transformed into *E. coli* strain JM109 according to manufacturer’s instructions for the QuikChange II Site-Directed Mutagenesis kit substituting JM109 for XL-1Blue cells (**Table 1**) and the transformants were selected on LB agar supplemented with ampicillin (100 μg/mL). After plasmid purification from selected transformants with QIAprep Spin Miniprep kit (Qiagen), the specific mutation was confirmed by DNA sequencing as with the sequencing of pQE::*Cj0355c.* This mutated version of CosR was named rCosRC218S and was purified from JM109pQE::*Cj0355c*652 using the same method as with rCosRWT.

The recombinant CmeR, named rCmeRSS, was induced and purified from JM109pQECmeRSS (**Table 2**) under native conditions as described in the manufacturer’s instruction (Qiagen). Construction of this strain was described previously in Oakland et al. (2011). rCmeRSS was purified using the same method as rCosRWT and rCosRC218S.

### Electrophoretic Mobility Shift Assays (EMSA)

Electrophoretic mobility shift assays was used to assess binding of CosR to the *cmeABC* promoter or its derivatives. Primers GSF and GSR1 (Lin et al., 2005a) (**Table 3**) were used to amplify a 170-bp region of the *cmeABC* promoter from strains 81–176 (named 81–176 promoter), NCTC 11168 (named 11168 promoter), and X7199 (named X7199 promoter) as described previously (Grinnage-Pulley and Zhang, 2015) (**Table 1**). An internal fragment of *cmeA* was amplified with primers AF and AR (**Table 3**) and was used as a negative control probe (Lin et al., 2005a). A 14-base pair deletion between the CosR and CmeR binding sites of *cmeABC* was designed based on the sequence of 81–176 and this probe was named P14D. A pUC57 vector carrying the P14D sequence was synthesized (Genscript) and then amplified using the GSF and GSR1 primers (Lin et al., 2005a) (**Table 3**). All probes were purified with QIAquick PCR purification kit and labeled with DIG-11-dd-UTP (Roche) as described previously (Lin et al., 2005a).

To confirm the binding specificity of CosR to the *cmeABC* promoter before and after cysteine mutation, the 11168 promoter probe or the *cmeA* probe (negative binding control; 0.05 pmol) were mixed with 250 ng of rCosRWT or rCosRC218S in the reaction buffer (14.4 μL total) according to the method of Alekshun et al. (2000) and Lin et al. (2005a). A control reaction was prepared with the 11168 promoter probe without addition of rCosRWT or rCosRC218S. Reactions were incubated for 30 min at room temperature. Promega DNA loading buffer was added to each reaction and then the reaction was run at 200 V for 45 min on a 6% polyacrylamide gel in 0.25X TBE buffer. Transfer to a positively charged membrane by vacuum and detection of DIG with CDP Star (Roche) were performed as previously described (Lin et al., 2005a).

To assess the effect of oxidation on binding of CosR to the *cmeABC* promoter, the 11168 promoter probe (0.05 pmol) was mixed with 250 ng of rCosRWT or rCosRC218S, incubated for 30 min at room temperature, and then hydrogen peroxide was added to the reactions at final concentrations of 0, 5, 10, or 20 nM (final volume 14.4 μL). Reactions were incubated for an additional 30 min at room temperature. Electrophoresis, transfer, and detection were performed as described above.

To determine if CosR and CmeR interfere with each other in binding to the *cmeABC* promoter, dual binding EMSA assays were performed using the promoter DNA probes that had varied lengths of spacing between the CmeR-binding site and the CosR-binding site. Dual binding of CosR and CmeR utilized 81–176 *cmeABC* promoter probe which has 17 base pairs (bp) between the CmeR and CosR binding site as a full length promoter control. The second probe containing a reduced distance between the CmeR and CosR binding sites was either the promoter probe of X7199, which has a 5 bp deletion, or probe P14D with a 14 bp deletion. Each probe (0.05 pmol) was incubated with 200 ng of rCmeRSS alone, 400 ng of rCosRC218S alone, or both rCmeRSS and rCosRC218S at 200 and 400 ng, respectively, in the reaction mixture (14.4 μL). A control reaction was prepared for each probe without the protein. Reactions were incubated at room temperature for 30 min. Promega DNA loading buffer was added to each reaction, which was then run at 200 V for 55 min on a 6% polyacrylamide gel in 0.25X TBE buffer. Transfer and detection were performed as described above.

### Transcriptional Fusion and β-Galactosidase Assay

Various *cmeABC* promoters were fused to the promoter-less *lacZ* gene in pMW10 (Wösten et al., 1998). Construction of strains 81–176 and 81–176Δ*cmeR* containing the plasmids pMW10, pMW11168, pMW81–176, and pMWX7199 was described previously (Grinnage-Pulley and Zhang, 2015) (**Table 2**). Plasmids pMW11168, pMW81–176, and pMWX7199 contained the *cmeABC* promoter from NCTC 11168, 81–176, and X7199, respectively, fused to the *lacZ* reporter gene, while pMW10 contains the reporter gene, *lacZ*, without a fused promoter. Plasmid pMW561 carries the *Cj0561c* promoter from strain NCTC 11168. *Cj0561c* is repressed by CmeR, not CosR, serving as a negative control for CosR regulation. pMW561 (Guo et al., 2008) (**Table 2**) was extracted from 11168W7pMW561 (Guo et al., 2008) (**Table 1**) using the QIAprep Spin Miniprep kit and transformed into DH5α via heat shock as described by Miller (1992). Tri-parental mating with DH5αpMW561 and DH5αpRK2013 was used to transfer the plasmid into *C. jejuni* strains 81–176 or 81–176Δ*cmeR* according to the method of Miller et al. (2000).

Overnight cultures of 81–176 or 81–176Δ*cmeR* with pMW10 (Wösten et al., 1998), pMW11168, pMW81–176, pMWX7199, or pMW561 were grown on MH agar supplemented with kanamycin (30 μg/mL) and then harvested in MH broth with kanamycin (30 μg/mL). As *cosR* is an essential gene and cannot be knocked out, inhibition of *cosR* was performed with the anti-*cosR* peptide nucleic acid (PNA) at 1.5 μM, a level that decreased CosR levels, but did not inhibit *C. jejuni* growth as reported by Hwang et al. (2011). The PNA (KFFKFFKFFK-O-CATTTGTTCTATCCTT) (Hwang et al., 2011) was obtained from PNA Bio, Inc. PNA is a synthetic DNA mimic with a polyamine backbone able to bind to complementary DNA or RNA following Watson–Crick binding principles (Nielsen et al., 1991; Egholm et al., 1993). Once inside bacterial cells, PNA inhibits gene expression in a target-specific manner (Good and Nielsen, 1998; Larsen et al., 1999; Kaihatsu et al., 2004). Western blotting was used to confirm that the anti-CosR PNA indeed inhibited expression of CosR. The harvested cultures were adjusted in MH-kanamycin broth to OD_600_ ∼0.07 and aliquoted to two tubes. The first tube was incubated with 1.5 μM anti-*cosR*-PNA and the second was incubated without the anti-*cosR*-PNA. All tubes were incubated by shaking at 180 rpm for 8 h at 42°C under microaerobic conditions (Hwang et al., 2011). β-Galactosidase assays were performed in triplicate samples for three independent experiments (Miller, 1992). Means were calculated for each promoter-condition combination.

The repressive effects (fold changes) of CosR and CmeR on the *cmeABC* promoter were calculated as follows based on the mean Miller units (**Table 4**). The individual effect of CosR was calculated as the relative fold change in transcription levels in the 81–176 wild-type background with and without the anti-*cosR*-PNA. The individual effect of CmeR was calculated as the relative fold change in transcription between the 81–176Δ*cmeR* background and the 81–176 wild-type background without the anti-*cosR* PNA. The sum effect of CosR and CmeR was calculated as the relative fold change in transcription levels between the 81–176Δ*cmeR* background with the anti-*cosR*-PNA and the 81–176 wild-type background without the anti-*cosR*-PNA. The repressive effects for each regulator were statistically analyzed after log2 transformation of the transcriptional data and one-way analysis of variance (ANOVA) in SAS (version 9.3). The comparisons of the transcriptional activity for each promoter, 11168, 81–176, and X7199, under dual repression by CosR and CmeR were calculated as follows based on the mean Miller units (**Table 5**). Mean transcriptional activity was first calculated for each promoter in the 81–176 wild-type background without the anti-*cosR*-PNA. Fold changes were calculated by the transcription of the X7199 promoter over either the 81–176 or 11168 promoter and the transcription of 81–176 promoter over the 11168 promoter. Statistical analysis for the comparisons were was performed using Student’s *t*-test with Welch’s corrections (GraphPad InStat^®^ Version 3.06).

**Table 4:** Repressive effects of CosR and CmeR on transcription of *cmeABC.*

Promoter	Individual Effect of CmeR^1^	Individual Effect of CosR^2^	Sum Effect of CosR and CmeR^3^
11168	4.6^∗^	1.8^∗^	6.3^∗^
81–176	3.7^∗^	2.1^∗^	4.9^∗^
X7199	2.6^∗^	1.7^∗^	3.5^∗^

*^∗^Indicates significance (*p* < 0.05) in effect for each promoter. ^1^Calculated as the relative fold change in transcription levels of the 81–176Δ*cmeR* background without the anti-*cosR* PNA over the transcription level of the 81–176 wild-type background without the anti-*cosR* PNA. ^2^Calculated as the relative fold change in transcription levels of the 81–176 wild-type background with the anti-*cosR*-PNA over the transcription levels in the 81–176 wild-type background without the anti-*cosR*-PNA. ^3^Calculated as the relative fold change in transcription levels of the 81–176Δ*cmeR* background with the anti-*cosR*-PNA over the transcription levels of the between the 81–176 wild-type background without the anti-*cosR*-PNA.*

**Table 5:** *cmeABC* promoter activities compared under dual repression by CosR and CmeR.

Promoter comparison	Spacer length (base pairs)	Fold change^1^	Cause of difference
X7199 to 11168	12 vs. 17	2.9^∗^	Spacer or CmeR
81–176 to 11168	17	1.4	None
X7199 to 81–176	12 vs. 17	2.1^∗^	Spacer length

*^∗^Indicates a significant difference (*p* < 0.05) between promoters. ^1^Calculated using the transcription level of each promoter in the wild-type 81–176 background without the anti-*cosR* PNA.*

## Results

### Oxidation of C218 in CosR Reduced *cmeABC* Promoter Binding

Two recombinant CosR (rCosR) proteins, rCosRWT, and rCosRC218S, were evaluated for their ability to bind the *cmeABC* promoter. The two proteins differed in one amino acid: cysteine 218 (C218) in the wild-type protein (rCosRWT) was replaced by serine in the mutant protein (rCosRC218S). To confirm that mutation of C218 did not affect binding specificity of CosR, EMSA was performed with the 11168 promoter probe incubated with rCosRWT or rCosRC218S. Additionally, the proteins were incubated with an internal *cmeA* fragment as a negative control for specificity. Both proteins bound specifically to the *cmeABC* promoter, forming DNA-protein complexes, and did not bind to the internal *cmeA* fragment (data not shown), consistent with the finding that CosR specifically interacts with the promoter of *cmeABC* as described by Hwang et al. (2012). This result indicates that the cysteine mutation did not alter the binding specificity.

**FIGURE 1:**
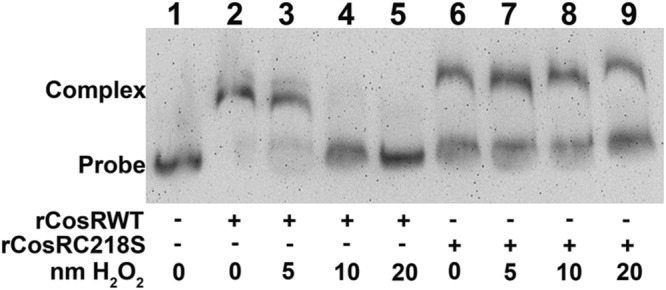
**Sensitivity of CosR to oxidation by hydrogen peroxide.** Binding of 250 ng of rCosRWT (lanes 2–5) or rCosRC218S (lanes 6–9) to DIG-labeled 11168 *cmeABC* promoter DNA (0.05 pmol) by EMSA. No protein was added to lane 1 as a probe-only control. The promoter probe and protein were incubated for 30 min prior to addition of hydrogen peroxide. Hydrogen peroxide was added for final concentrations of 0 nM (lanes 2 and 6), 5 nM (lanes 3 and 7), 10 nM (lanes 4 and 8), or 20 nM (lanes 5 and 9). After addition of hydrogen peroxide, all reactions were incubated for an additional 30 min before electrophoresis.

To evaluate the role of cysteine in CosR binding activity under oxidative stress, the rCosRWT and rCosRC218S proteins were treated with hydrogen peroxide and then analyzed by EMSA. Cysteine residues are known to be sensitive to oxidation, while serine is not (Antelmann and Helmann, 2011). As shown in **Figure 1**, binding of the *cmeABC* promoter by rCosRWT decreased as hydrogen peroxide concentration increased (lanes 2–5). At 20 nM of hydrogen peroxide (**Figure 1**, lane 5), the binding of rCosRWT to the DNA probe was completely inhibited and the unbound probe was at the level of the free probe control (**Figure 1**, lane 1). In contrast, the promoter binding by rCosRC218S was not affected by treatment with hydrogen peroxide (**Figure 1**, lanes 6–9). This indicated that the C218 in CosR was sensitive to hydrogen peroxide and oxidation of this residue interfered with CosR binding to promoter DNA.

### Dual Binding of the *cmeABC* Promoter by CosR and CmeR

**FIGURE 2:**
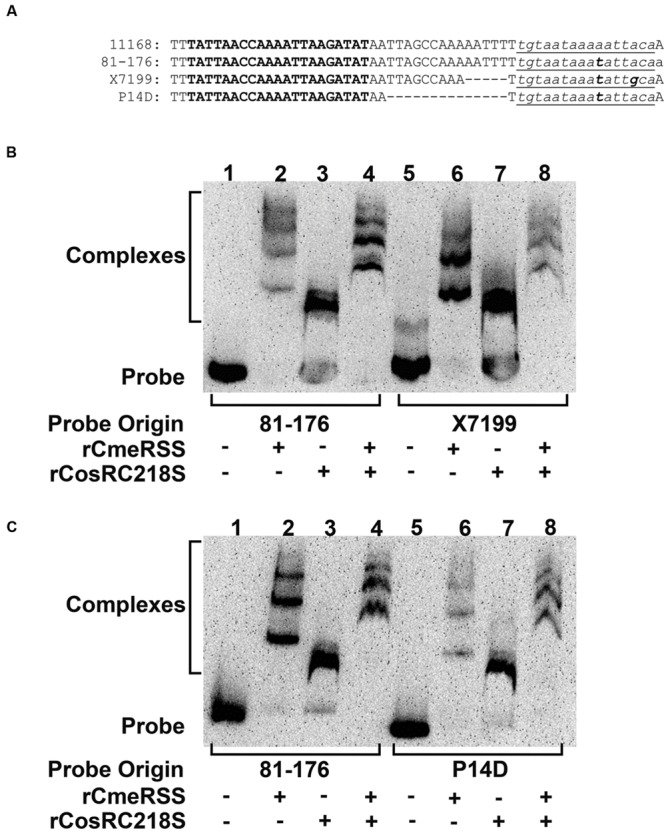
**Dual binding of CosR and CmeR to variants of the *cmeABC* promoter sequence. (A)** Alignment of *cmeABC* promoter sequences from strains NCTC 11168 (11168), 81–176, and X7199, along with the *in silico* designed P14D probe. The CosR binding site is indicated in bold and the CmeR binding site indicated in lowercase, underlined italics. Mutations in the CmeR binding site are indicated in bold, lowercase, underlined italics. (–) indicates a deleted base. **(B)** EMSA of the *cmeABC* promoter probes from 81–176 (lanes 1–4) and X7199 (lanes 5–8) incubated with 200 ng of rCmeRSS (lanes 2 and 6), 400 ng of rCosRC218S (lanes 3 and 7), or both proteins (lanes 4 and 8). No protein was added to lanes 1 and 5 as probe-only controls. **(C)** EMSA of the *cmeABC* promoter probes from 81 to 176 (lanes 1–4) and P14D (lanes 5–8) incubated with rCmeRSS, rCosRC218S (lanes 3 and 7), or both proteins as in **(B)**. No protein was added to lanes 1 and 5 as probe-only controls. All promoter probes were 0.05 pmol per reaction.

The *cmeABC* promoter contains binding sites for both CmeR and CosR (Hwang et al., 2011, 2012) (**Figure 2A**). To determine if binding by one protein interferes with concurrent binding by the second, promoter sequences with various lengths between the two binding sites were used as probes in EMSA. Both the 11168 and 81–176 *cmeABC* promoter probes had 17 base pairs (bp) between the CosR and CmeR binding sites (**Figure 2A**). The *cmeABC* promoter of strain X1799, isolated from a human, was previously sequenced during a screening of *C. jejuni* isolates for CmeABC expression (Grinnage-Pulley and Zhang, 2015) and was found to have a reduced distance of 12 bp between the CosR and CmeR binding sites (**Figure 2A**). To further reduce the distance between regulator binding sites without altering either the CosR or CmeR binding site sequence, the P14D probe containing only 3 bp between the binding sites was synthesized (**Figure 2A**). Each promoter probe was incubated with rCmeRSS or rCosRC218S individually or in combination. rCosRC218S was used to ensure that no alteration in binding would occur due to the aerobic conditions of the *in vitro* testing.

Evaluation of individual protein binding showed that rCosRC218S bound equally well to the 81–176 (**Figures 2B,C**, lane 3), X7199 (**Figure 2B**, lane 7), and P14D (**Figure 2C**, lane 7) promoter probes. However, binding of rCmeRSS to the promoter probes varied (**Figures 2B,C**, lanes 2 and 6). Specifically, compared to the other probes, rCmeRSS binding to the P14D probe was much weaker, yielding three light bands (**Figure 2C**, lane 6), which suggests reduced interaction between the protein and the P14D. This indicated that the space between the CosR and CmeR binding sites in the *cmeABC* promoter affected the binding by CmeR, but not by CosR.

Evaluation for dual binding by rCosRC218S and rCmeRSS by adding the proteins in combination to promoter probes demonstrated simultaneous binding (**Figure 2**). The protein combination of rCosRC218S and rCmeRSS incubated with the 81–176 promoter probe produced three dark bands (**Figures 2B,C**, lane 4). Noticeably, the lowest band of this triplet (lane 4) was higher than the single rCosRC218S band (**Figures 2B,C**, lane 3) and the lowest band of rCmeRSS (**Figures 2B,C**, lane 2). The other two bands when rCosC218S and rCmeRSS (**Figures 2B,C**, lane 7) were incubated with the probe were also higher than the top bands of rCmeR-probe combination (lane 2). Incubation of rCosRC218S and rCmeR with the X7199 promoter probe also produced three bands (**Figure 2B**, lane 8), but their intensities were much weaker than the 81–176 probe-dual protein complex bands (**Figure 2B**, lane 4). Similarly, incubation of the two proteins with P14D also yielded bands, but weaker (**Figure 2C**, lane 8) compared to the 81–176 probe-dual protein bands (**Figure 2C**, lane 4). These shifted bands in lanes 4 and 8 suggest both CosR and CmeR bound to the probes simultaneously. If rCmeRSS prevented rCosRC218 binding or vice versa, we would expect four bands with the lowest representing rCosRC218S binding and the upper triplets representing rCmeRSS binding. The weaker band intensities in the presence of both proteins (**Figures 2B,C**, lane 8) indicated reduced binding to the X7199 and P14 probes. Binding was not affected by the order of proteins added to the probes (data not shown). Together, these results suggested that reducing the distance between the CosR and CmeR binding sites in the *cmeABC* promoter interfered with dual binding by the regulatory proteins.

### Dual Regulation of *cmeABC* by CmeR and CosR *In Vivo*

**FIGURE 3:**
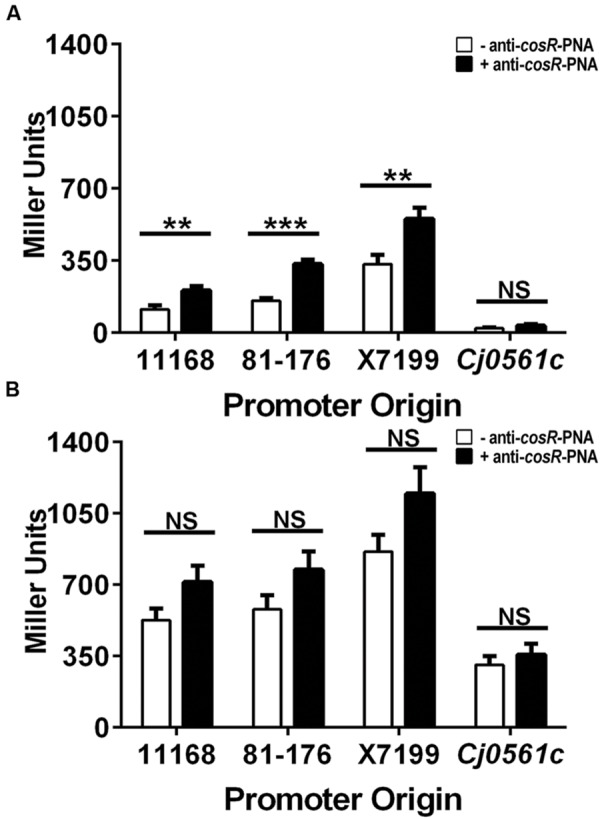
**Effect of CosR inhibition by anti-*cosR*-PNA on transcription from the *cmeABC* promoter in the presence or absence of CmeR.** Expression from 11168, 81–176, or X7199 *cmeABC* promoters or the *Cj0561c* promoter (561) in β-galactosidase assays. Presence or absence of CmeR was determined by using 81–176 wild-type **(A)** and 81–176Δ*cmeR* **(B)** for the transcriptional fusions. Cultures were incubated with (+ anti-*cosR*-PNA) or without (- anti-*CosR*-PNA) 1.5 μM of the anti-*cosR-*PNA. The *Cj0561c* promoter was a control for regulation solely by CmeR, not by CosR. Data represents +means with standard error from three independent experiments. Unpaired Student’s *t*-test with Welch’s correction was used for comparison. ^∗∗^*p* < 0.01 and ^∗∗∗^*p* < 0.001, NS, not significant; *p* > 0.05 of the means with significance set at 0.05.

Based on EMSA, CosR and CmeR can bind to the *cmeABC* promoter simultaneously, constituting a dual mechanism for regulating expression of *cmeABC*. To quantify the effects of these regulators in modulating *cmeABC* expression, *in vivo* transcriptional fusion assays were performed. Plasmids containing the *cmeABC* promoter from strains NCTC 11168 (11168 promoter), 81–176 (81–176 promoter), or X7199 (X7199 promoter; **Figure 2A**) were fused to a promoterless *lacZ* gene. These plasmids were transferred into wild-type 81–176 (**Figure 3A**) to evaluate expression in the presence of CosR and CmeR and into 81–176Δ*cmeR* to evaluate expression in the absence of CmeR (**Figure 3B**). Since *cosR* is an essential gene and cannot be inactivated in *Campylobacter*, we used anti-*cosR* PNA (**Figure 3**, black bars) to assess the effect of CosR inhibition on *cmeABC* expression. This was performed in both the 81–176 background to demonstrate the effect of CmeR on *cmeABC* expression when CosR levels were reduced and in 81–176Δ*cmeR* background to demonstrate the effect of both the absence of CmeR and reduction of CosR on *cmeABC* expression. Western blotting demonstrated the anti-*cosR* PNA at 1.5 μM resulted in approximately twofold reduction in CosR expression (data not shown), consistent with the result reported by Hwang et al. (2011).

In the presence of both CmeR and CosR (**Figure 3A**, open bars), transcription was the highest from the X7199 promoter. This is consistent with the results reported in our previous study (Grinnage-Pulley and Zhang, 2015). Inhibiting CosR in wild-type 81–176 with the anti-*cosR* PNA caused a significant (*p* < 0.05) increase in transcription of all promoters (**Figure 3A**, solid bars): 1.8-, 2.1-, and 1.7-fold for the 11168, 81–176, and X7199 promoters, respectively (**Table 4**). This ratio of transcription in 81–176 wild-type treated with the anti-*cosR* PNA to untreated was defined as the individual effect of CosR. Inhibiting CosR in the 81–176Δ*cmeR* background (**Figure 3B**) caused further increase in the transcription from all *cmeABC* promoters compared to the non-treated controls, but the increase was not statistically significant at 1.4-fold for both the 11168 and 81–176 promoters and 1.3-fold for the X7199 promoter. This indicated that CosR functions as a repressor for *cmeABC* in the presence of CmeR and confirmed the results of the EMSA. As a control for the specific effect of the anti-*cosR* PNA, the *Cj0561c* promoter was also fused to the promoterless *lacZ* gene. *Cj0561c* is known to be repressed by CmeR (Guo et al., 2008; Dzieciol et al., 2011), not by CosR. Transcriptional level from the *Cj0561c* promoter was not influenced by the anti-*cosR* PNA either in the wild-type 81–176 background (**Figure 3A**) or in the 81–176Δ*cmeR* background (**Figure 3B**), indicating the specificity of CosR to the *cmeABC* promoter. However, transcription from the *Cj0561c* promoter showed a significant (*p* < 0.01) increase in 81–176Δ*cmeR* compared with the wild-type 81–176, which is consistent with the known regulation of *Cj0561c* by CmeR (Guo et al., 2008).

The individual effect of CmeR on *cmeABC* transcription was also quantified (**Table 4**). The individual effect of CmeR was defined as the ratio of transcription in the 81–176Δ*cmeR* background without the anti-*cosR* PNA (**Figure 3B**, open bars) to the 81–176 wild-type background the without anti-*cosR* PNA (**Figure 3A**, open bars). Loss of CmeR, with CosR present caused significant (*p* < 0.05*)* increases in *cmeABC* transcription of 4.6-fold for the 11168 promoter, 3.7-fold for the 81–176 promoter, and 2.6-fold for the X7199 promoter (**Table 4**). Anti-*cosR*-PNA treatment in the 81–176Δ*cmeR* background also had a significant (*p* < 0.05*)* effect on *cmeABC* transcription with 3.5-, 2.3-, and 2.1-fold increases for the 11168, 81–176, and X7199 promoters, respectively, compared to the 81–176 wild-type background treated with *anti-cosR* PNA. These effects confirmed that CmeR functions independently of CosR. Additionally, the lower magnitude of *cmeABC* inhibition by CosR compared with CmeR suggested that CmeR functioned as a primary regulator for *cmeABC* and CosR as a secondary regulator for *cmeABC*.

### Shortened Spacer Length Reduces CmeR Binding

The length of the spacer between CosR and CmeR binding sites in the *cmeABC* promoter were shown to modulate dual binding by the proteins by EMSA (**Figure 2**). To quantify the effect of spacer length on dual binding, transcription under dual repression was assessed. This value, the sum effect of CosR and CmeR (**Table 4**), was the ratio of transcription in the 81–176Δ*cmeR* background with the anti-*cosR*-PNA (**Figure 3B**, solid bars) to the untreated, 81–176 wild-type background (**Figure 3A**, open bars). Based on the sum effect, *cmeABC* transcription was significantly (*p* < 0.05) increased by 6.3-, 4.9-, and 3.5- fold for the 11168, 81–176, and X7199 promoters, respectively (**Table 4**). As the promoters contained mutations in the CmeR binding site or the spacer region, but not in the CosR binding site, the transcription from different promoters in the wild-type background (*cmeABC* is under dual repression by CmeR and CosR) was further compared (**Table 5**). Transcription from the shortened X7199 promoter showed a significant 2.9-fold (*p* < 0.01) increase over the full-length 11168 promoter (**Table 5**). In contrast, the full-length promoters, 81–176 to 11168 had a non-significant 1.4-fold increase. To confirm the larger increase for the X7199 promoter was not solely due to the CmeR binding site mutations of the X7199 promoter, transcription from the X7199 promoter was compared to the 81–176 promoter, which shared a point mutation in the CmeR binding site with X7199 (**Figure 2A**). This comparison showed a significant (*p* < 0.05) 2.1-fold increase (**Table 5**). Thus, even with mutations in the CmeR binding site, the X7199 was less repressed by CmeR under dual regulation. Taken together, these results indicated that reduction of the spacer length between the binding sites decreased binding by CmeR during dual repression of *cmeABC* and lead to increased *cmeABC* levels.

## Discussion

The multidrug eﬄux pump CmeABC is well known for its roles in antimicrobial resistance and bile resistance (Lin et al., 2002, 2003, 2005b; Pumbwe et al., 2004; Cagliero et al., 2005; Ge et al., 2005; Yan et al., 2006; Gibreel et al., 2007). This eﬄux system is under negative regulation by CmeR (Lin et al., 2005a,b; Cagliero et al., 2007; Gu et al., 2007; Guo et al., 2008; Lei et al., 2011; Shen et al., 2011). More recently, CosR, an oxidative stress response regulator, was also found to modulate the expression of *cmeABC* (Hwang et al., 2012). In our study we demonstrate that CosR and CmeR bind simultaneously to the promoter sequence of *cmeABC* functioning as a dual regulatory mechanism for this eﬄux pump. During dual binding to the promoter, CosR and CmeR binding is independent of each other, but is affected by the length of the space between the two binding sites. In addition, we found that the sole cysteine (C218) of CosR is sensitive to cysteine oxidation; it may serve as a mechanism for CosR to sense oxidative stress. These findings provide new information on the complex regulatory mechanisms for CmeABC and the diverse signals that may modulate the expression and function of the predominant eﬄux system in *C. jejuni.*

Both CmeR and CosR have a specific binding site in the *cmeABC* promoter (Lin et al., 2005a; Hwang et al., 2011, 2012). CosR was described to recognize a 21 base binding site, ttaAanAaAAaTtAtagaTTt, which occurs in multiple promoters including *cmeABC* (Hwang et al., 2011). In the *cmeABC* promoter, the CosR binding site is 17 bases upstream of the CmeR binding site (Lin et al., 2005a; Hwang et al., 2012). Despite the proximity of the two repressor binding sites, CosR was shown to specifically recognize the CosR binding sequence (Hwang et al., 2012) (**Figure 2**). The inability of CosR to influence *Cj0561c* expression as measured by transcriptional fusion (**Figure 3**) was correlated with the lack of the CosR specific binding site within the *Cj0561c* promoter. A similar situation occurs in the *katA* promoter, with multiple regulatory protein binding sites. The *katA* promoter contains binding sites for PerR and holo-Fur regulators in addition to the CosR binding site (Butcher et al., 2012; Hwang et al., 2012). CosR positively regulates *katA*, but *katA* is negatively regulated by PerR and Fur, peroxide and iron responsive regulators (van Vliet et al., 1998, 1999; Palyada et al., 2009; Kim et al., 2011). The specific interaction of CosR with its binding site within the *katA promoter* was demonstrated by DNA footprinting (Hwang et al., 2012). CosR binds to the CosR-specific binding site in the *katA* promoter, which does not overlap with the sites for other regulators (Hwang et al., 2012), similar to the CosR and CmeR binding sites in the promoter of *cmeABC.*

Dual binding of the *cmeABC* promoter by CosR and CmeR, demonstrated *in vitro* by EMSA (**Figure 2**), and dual regulation *in vivo* (in *Campylobacter*) confirmed using transcriptional assays (**Figure 3**), were influenced by the distance between the two binding sites. The NCTC 11168 and 81–176 *cmeABC* promoters have a full length 17 bp spacer between the CosR and CmeR binding sites and have similar sequences except for an A to T substitution in the CmeR binding site of the 81–176 promoter (Lin et al., 2002). Both the X7199 and P14D promoters, with 12 and 3 bp spacers, respectively, showed decreased DNA-protein complexes during dual binding with the greatest binding reduction in P14D (**Figures 2A,B**; lane 8). Consistently, this spacer reduction increased *cmeABC* transcription under dual regulation as the sum effect of CmeR and CosR was lowest for the X7199 promoter (**Table 4**). Notably, shortening the spacer sequence did not affect individual binding of CosR for the X7199 and P14D promoters on EMSA (**Figures 2B,C**, lane 7) and X7199 in transcriptional fusion assays (**Figure 3**). This suggests that CosR is not affected in dual binding by the shortened spacer in the promoter. It should be pointed that the EMSA technique used in this study has limitation in quantitative measurement of the binding affinity of each regulator to the *cmeABC* promoter DNA, which can be measured by determining the dissociation constant and will be pursued in future studies.

The artificially designed DNA probe, P14D (**Figure 2A**) also had reduced binding when incubated with CmeR alone (**Figure 2C**), further indicting altered CmeR binding when the region upstream of the CmeR binding site is altered. However, the X7199 promoter used in transcriptional assays had a substitution in the CmeR binding site. This same substitution is also found in the full length 81–176 promoter as well. Yet transcription from the X7199 promoter is still significantly higher than the 81–176 promoter under dual binding (**Table 5**), suggesting that the effect on CmeR binding was mainly due to the binding site mutation. Based on these findings, we speculated that the spacer sequence facilitates CmeR binding to its binding site, but is not required for CosR to interact with its binding site. We speculate dual binding with a reduced spacer creates steric hindrance for CmeR, resulting in reduced binding by CmeR and consequently enhanced transcription of *cmeABC* (**Figures 2** and **3**).

The observed magnitude of CosR repression on *cmeABC* was lower than that of CmeR, suggesting CosR functions as a secondary regulator for *cmeABC*. This finding is based on PNA inhibition of *cosR*, which did not completely abolish CosR. The inhibition of CosR represents a limitation of this study as CosR is essential for *C. jejuni* (Hwang et al., 2011) and its gene could not be deleted. Without a way to delete the gene, the absolute magnitude of CosR inhibition on *cmeABC* expression cannot be measured. Thus the individual and combined effects of the regulation should be interpreted cautiously.

Many genes in the CosR regulon are involved in the oxidative stress response (Hwang et al., 2011, 2012). CosR positively regulates *katA* and *ahpC*, but negatively regulates others genes such as *sodB, dps*, and *cmeABC* (Hwang et al., 2011, 2012). Under oxidative stress, reactive oxygen species (ROS) cause oxidative damage to cellular components, reducing growth, and at high levels, can cause cell death. Thus, CosR-mediated response and defense against oxidative stress is important for *Campylobacter* physiology. Although the role of CosR has been defined, how it senses oxidative stress is not known. Examination of the CosR sequence identified a single cysteine residue, C218, which is predicted to be localized at the dimer face. Cysteine residues are known sites subject to modification by ROS, reactive nitrogen species, and reactive electrophilic species and are involved in redox sensing by many regulatory proteins (Chen et al., 2006, 2008; Antelmann and Helmann, 2011; Dubbs and Mongkolsuk, 2012). Cysteine oxidation can result in disulfide bond formation, which often alters protein conformation and modulates DNA binding activity (Antelmann and Helmann, 2011). OxyR, MgrA, AsrR, and MexR are regulators that utilize cysteine oxidation as a mechanism to regulate DNA binding, and are also regulators of eﬄux pumps (Truong-Bolduc et al., 2005; Chen et al., 2008; Antelmann and Helmann, 2011; Lebreton et al., 2012). In this study, we demonstrated that C218 in CosR is sensitive to oxidative stress and oxidation of this cysteine disassociated CosR from the *cmeABC* promoter (**Figures 1** and **2**, lanes 1–5) as demonstrated by EMSA. This suggests that cysteine modification may affect the function of CosR. This conclusion is further supported by findings in other studies, in which expression of *cmeABC* was induced under oxidative stress (Hwang et al., 2012). As CosR modulates the expression of multiple genes in *C. jejuni* (Hwang et al., 2012), altered function of CosR by cysteine modification may affect the expression of genes involved in multiple pathways. However, this possibility is required to be determined *in vivo* (within *Campylobacter*). Since *cosR* is an essential gene and may not be mutated with loss of its function, a novel strategy is needed to assess the specific role of C218 in sensing oxidative stress and modulating CosR function in *Campylobacter*, which will be pursued in future studies.

In addition to CmeABC, several members of the resistance-nodulation-cell division (RND) eﬄux family, such as MexAB-OprM of *Pseudomonas aeruginosa* and AcrAB-TolC of *Salmonella enterica* serovar Typhimurium and *Escherichia coli* are also regulated by multiple regulators and signals (Evans et al., 2001; Saito et al., 2001; Chen et al., 2008; Nikaido et al., 2011). In *P. aeruginosa* the MexAB-OprM eﬄux pump is a negatively regulated by MexR (Evans et al., 2001; Saito et al., 2001). MexR senses oxidative stress (e.g., hydrogen peroxide and cumene-hydrogen peroxide) by oxidation of its two cysteine residues, leading to overexpression of MexAB (Chen et al., 2008). MexAB is also negatively regulated by NalD, a TetR family repressor (Morita et al., 2006). NalD binding to novobiocin releases repression of MexAB, resulting in its overexpression (Chen et al., 2016). The AcrAB-TolC is an archetype RND pump found in multiple bacterial species. In both *E. coli* and *S. typhimurium*, AcrAB-TolC is positively regulated by MarA, Rob, and SoxS (also by RamA in *Salmonella*) and negatively regulated by AcrR (Ma et al., 1996; Nikaido et al., 2008, 2011; Duval and Lister, 2013). Induction by bile is mediated by RamA in *S. typhimurium* and by Rob in *E. coli*, while the oxidative response in both organisms is mediated by SoxS (Greenberg et al., 1990; Rosenberg et al., 2003; Nikaido et al., 2008, 2011; Duval and Lister, 2013). SoxS is induced by the superoxide generator paraquat and up-regulates AcrAB expression (Greenberg et al., 1990; Nikaido et al., 2011; Duval and Lister, 2013). Together, these examples illustrate the complex regulation of RND-type eﬄux systems by both local and global regulators that respond to different stimuli.

In summary, the dual regulation of *cmeABC* by CosR and CmeR suggests that this predominant eﬄux transport system is capable of responding to different signals. CosR may sense and respond to oxidative stress, while CmeR responds to bile (Lin et al., 2005b; Lei et al., 2011), salicylate (Shen et al., 2011), and possibly other unidentified compounds. As a microaerophilic zoonotic pathogen prevalent in food producing animals, *Campylobacter* frequently encounters environmental stresses such as antimicrobials, bile, and oxidative challenges (Begley et al., 2005; Corcionivoschi et al., 2012). *Campylobacter* utilizes multiple mechanisms for environmental adaptation, but CmeABC is a key player for antibiotic resistance and intestinal colonization by mediating resistance to antimicrobials and bile (Lin et al., 2003, 2005a,b; Guo et al., 2008). This study suggests an additional role of CmeABC in oxidative stress response via a CosR-mediated mechanism. How exactly CmeABC contributes to oxidative stress defense is unknown and remains to be examined in future studies. Nevertheless, the sophisticated mechanisms of regulation signify the importance of this eﬄux system in *Campylobacter* pathobiology and indicate that its functions are more diverse than previously expected. All together, these observations further justify CmeABC as a potential target for the development of anti-*Campylobacter* interventions.

## Author Contributions

TG-P designed and performed the experiments, interpreted data, conducted the statistical analysis, and wrote and revised the manuscript. YM and LD designed and constructed the pQE::*Cj0355c* plasmid for producing recombinant CosR. QZ designed the study, provided reagents and supplies, interpreted data, and revised the manuscript.

## Conflict of Interest Statement

The authors declare that the research was conducted in the absence of any commercial or financial relationships that could be construed as a potential conflict of interest.

## Disclaimer

The funders had no role in study design, data collection and analysis, decision to publish, or preparation of the manuscript. The content is solely the responsibility of the authors and does not necessarily represent the official views of the National Institute of Allergy and Infectious Disease, the National Institutes of Health, or the China Scholarship Council.
